# Patient safety and the widespread use of herbs and supplements

**DOI:** 10.3389/fphar.2014.00142

**Published:** 2014-06-12

**Authors:** Samantha M. Werner

**Affiliations:** Department of Anesthesiology, The Ohio State University Wexner Medical CenterColumbus, OH, USA

**Keywords:** herbs and supplments, patient safety, herb-drug interactions, self-diagnosis, perioperative management

Though herbs and supplements are included in the realm of alternative and non-traditional medical treatments, their use is common. Over 4 billion people, or 80% of the world's population, use herbal remedies as their source of primary care (Ekor, 2014). This wide use offers an explanation for the prevalent concomitant intake of herbal remedies and supplements with prescribed medications. Patients who ingest herbs and supplements while taking prescription medicine may be putting themselves at risk, especially if they are preparing to undergo a surgical procedure (Dickinson, 2014). As a routine part of preoperative care, physicians review patients' past medical history, based on patient reports, as well as any prescribed medications. Though some physicians ask patients if they are taking herbal medications or supplements, patients may not freely disclose the ingestion of “natural” supplements unless specified to do so (Goldstein et al., 2007).

There are numerous clinical concerns associated with potential herb-drug interactions perioperatively which include cardiac instability, electrolyte imbalances, prolonged bleeding, and excessive sedation (Gallo et al., 2014). Herbal medicines can affect absorption, metabolism, distribution, and excretion mechanisms when administered with prescription drugs, since drug-metabolizing enzymes may be induced or inhibited (Skalli et al., 2007).

For instance, *Forst f*. (Piperaceae), common name Kava, inhibits sodium and calcium channels, thereby directly decreasing vascular resistance and blood pressure. Kava exhibits dopaminergic antagonism, which may produce adverse neurologic effects and cause excessive perioperative sedation (Skalli et al., 2007). There is also a risk of dietary supplement-drug interaction, such as omega-3 polyunsaturated acids and some vitamins while taking antiplatelets or anticoagulants. This combination puts patients at a risk for bleeding, as may interactions between anticoagulants and vitamins A, E, and coenzyme Q10 (CoQ10) (Gallo et al., 2014), as well as vitamin K.

Excessive and unexpected intraoperative bleeding may occur in patients who consume herbs and supplements (Dickinson, 2014). This bleeding may be caused by products containing *Allium sativum* (garlic), *Ginkgo biloba* (gingko), *Zingiber officinale* (ginger), *Salvia miltiorrhiza* (danshen), *Panax ginseng* (ginseng), and *Hypericum perforatum* (St John's Wort) (Gallo et al., 2014). Yet other herbs and supplements can prolong or counteract the effects of anesthesia and are likely caused by the modulation of gamma-aminobutyric acid (GABA) neurotransmission (Skalli et al., 2007).

A pharmacovigilence center at the University of Florence, Italy gathered preoperative assessment data of 478 patients from three hospitals. Of those patients, 50% used an herbal remedy and 23% were exposed to a potentially harmful herb-drug interaction (Gallo et al., 2014). In another study following 299 Israeli inpatients, 25% of patients consumed herbal or dietary supplements and in 72% of these cases, the hospital team was unaware of the patient's herb or supplement intake. Patients cited reasons for not informing the treatment team of herb or supplement intake as “not important, not medicine, lack of physician understanding, or lack of physician inquiry” (Goldstein et al., 2007).

It should also be noted that many women have reported using herbs during pregnancy, while breastfeeding, and administering natural remedies to children with the belief that herbs and supplements are safer than chemical drugs (Skalli et al., 2007). Strategic advertising tactics from the multi-billion dollar nutraceutical industry have paved the way for consumers to cling to the notion that natural is synonymous with safe (Skalli et al., 2007). In the same way that pharmaceuticals are widely advertised to the public, nutraceuticals are made readily available by means of “insufficient health claims and aggressive marketing” (Leonti and Casu, 2013).

Herbs and supplements also have undesired effects, yet are governed by different regulations than their pharmaceutical counterparts. Though regulated by the FDA, supplements do not require approval to be sold, allowing the manufacturer to decide if their products are safe to sell or not (U. S. Food and Drug Administration, 2014). Herb and supplement regulations do not yet monitor the elimination of toxins in supplements, listing of contraindications, manufacturing techniques, product of origin, or chemical concentration (Skalli et al., 2007). Additionally, underreporting of adverse events associated with herb and supplement use remains an issue of concern (Gallo et al., 2014).

Many herbs and supplements have been studied in clinical trials, but much remains to be understood about their synergistic effects and many more supplements, in current use, have yet to be studied (Leonti and Casu, 2013). Due to these gaps in research, in addition to a lack of trust and communication between patients and physicians, patients may be taking natural supplements they deem to be innocuous and, instead, causing themselves unnecessary harm (Skalli et al., 2007; Williams Orlando, 2009; Dickinson, 2014). The analysis of herbal preparations must take into account multi-compound formulations that are synergistic and cannot be understood solely using reductionist means (Leonti and Casu, 2013).

Healthcare professionals need to routinely ask patients, if they are not already, what herbs and natural supplements they are consuming (Goldstein et al., 2007; Skalli et al., 2007; Gallo et al., 2014). Patients need to be informed that sharing supplement use with a healthcare provider may be instrumental in preserving health and avoiding adverse events, especially perioperatively (Dickinson, 2014). New research efforts to study both synergistic herbal blends and individual supplements, not yet studied but in current use, will contribute to the knowledge base that can help physicians and patients make informed health decisions (Skalli et al., 2007).

## Conflict of interest statement

The author declares that the research was conducted in the absence of any commercial or financial relationships that could be construed as a potential conflict of interest.
